# Clinical Quantitative Method Development of serum indoxyl sulfate (IS) assay using LC-MS/MS

**DOI:** 10.1016/j.bbrep.2025.102316

**Published:** 2025-10-24

**Authors:** Sohei Kobayashi, Kazuyuki Matsushita, Kenji Kawasaki, Masanori Seimiya

**Affiliations:** a Department of Medical Technology & Sciences, School of Health Sciences at Narita, International University of Health and Welfare, Chiba, Japan; b Department of Laboratory Medicine & Division of Clinical Genetics, Chiba University Hospital, Chiba, Japan

**Keywords:** Analytical validation, Clinical quantitative method development, Protein-bound uremic toxins (PBUT), Reference assay method, Chronic kidney disease (CKD)

## Abstract

**Background:**

Indoxyl sulfate (IS) accumulates in the blood with decreases in the voided volume due to a decline in renal function and promotes the progression of renal failure. Assay reagents for IS are marketed by several companies, but they have not been standardized because of the absence of a standard assay method. In this study, the performance of the LC-MS/MS (liquid chromatography tandem mass spectrometry) was evaluated for the establishment of a standard assay method.

**Methods:**

The same analytical procedure was performed by two examiners using the AB SCIEX QTRAP® 4500 LC-MS/MS System. A calibration curve was prepared according to the target analyte (IS)/internal standard: 3-Indoxyl sulfate-d4 potassium salt (IS-d4) peak area ratio, and the linearity, reproducibility, selectivity tests, interferent tests, limit of blank (LOB), limit of detection (LOD), lower limit of quantification (LLOQ), and correlations were evaluated.

**Results:**

By dilution tests of IS standard solution, linearity (R^2^ = 0.9995) passing through the origin was observed up to 440 μmol/L. Repeatability was coefficient of variation 2.6–4.7 % at 8.3–124.6 μmol/L, and the intermediate precision was 7.9–9.2 % at 12.9–171.2 μmol/L. In the selectivity testing, the recovery rate was 101.0–104.3 %. Regarding the effects of interferents, the interference rate was about 5.0 % at the maximum. LOB was 0.004 μmol/L, LOD was 1.248 μmol/L, and LOQ was 3.23 μmol/L. The correlation between this method (y) and conventional enzymatic analysis (x) was R = 0.996, y = 1.13x - 2.89.

**Discussion:**

The basic performance of this method was satisfactory. Also, because excellent precision was obtained in both examiners, the stability of the assay method was also suggested to be guaranteed. This method is considered useful as a reference method for IS assay.

## Highlights

• Assay reagents for Indoxyl sulfate (IS) are marketed by several companies, but they have not been standardized because of the absence.
• In this study, the performance of the LC-MS/MS was evaluated for the establishment of a standard assay method.
• The linearity, reproducibility, spike and recovery tests, interferent tests, limit of blank (LoB), limit of detection (LoD), lower limit of quantification (LLoQ), and correlations were evaluated.
• This method is considered useful as a reference method for indoxyl sulfate assay.

## Graphical abstract

**Figure:**
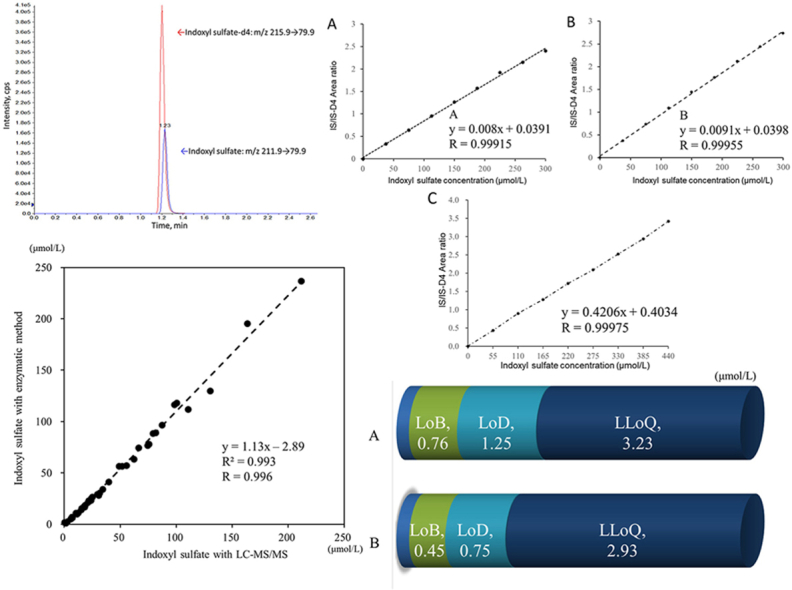

## 1 Introduction

Globally, chronic kidney disease (CKD) is estimated to affect 8–16 % of the population, and its prevalence increases with aging [1,2]. About 13 % of the Japanese adult population is estimated to suffer from CKD [3]. The concentrations of protein-bound uremic toxins (PBUT), such as indoxyl sulfate (IxS), p-cresyl sulfate (pCS), and p-cresyl glucuronide (pCG), are known as assessment markers for CKD [4]. Indoxyl sulfate (IS) is the metabolic end product of tryptophan, an amino acid. Tryptophan produced by protein degradation is converted to indole by the action of intestinal bacteria in the large intestine. As it is transported to the liver, it is metabolized to indoxyl, and IS is then produced by sulfuric acid conjugation [5]. It is excreted into the urine if the renal function is normal, but its circulating concentration increases with decreases in the voided volume or a decline in IS clearance capacity, if the renal function is impaired. IS accumulated in blood affects the whole body as a urinary toxin. The promotion of tissue fibrosis and induction of inflammation are known to be effects of IS, and they are related to exacerbation of kidney damage and aortic calcification in CKD [5,6]. It is also known to exert various adverse effects including vascular sclerosis and myocardial disorders, and its presence may also be strongly involved in the pathogenesis of coronary vascular disease (CVD) in CKD patients [7]. For these reasons, IS is needed as a biomarker for the evaluation of CKD and the introduction of hemodialysis. Accurate quantification of IS is essential for developing strategies for staging and monitoring CKD [5,8,9]. Although various methods have been reported to accurately quantify IS, enzymatic methods are common and practical at the clinical laboratory level. IS assay reagents are sold by multiple companies; however, since no standard assay method exists, they are limited to research use only and are not approved for clinical diagnostic applications [10]. This study was conducted to examine whether LC-MS/MS (liquid chromatography tandem mass spectrometry) has adequate performance as a reference assay method for IS by evaluating the linearity, precision, correlativity, selectivity tests, interferent tests, limit of blank (LOB), limit of detection (LOD), and lower limit of quantification (LLOQ). The originality of this study lies in conducting more comprehensive performance verification of standard measurement methods for IS than previous reports [[11], [12], [13]].

## 2 Materials and methods

### 2.1 Chemicals and reagents

Acetonitrile and formic acid for mass spectrometry were purchased from FUJIFILM Wako Pure Chemical Corporation (Osaka City, Osaka Prefecture, Japan). The IS standard solution used was indoxyl sulfate potassium salt purchased from Sigma-Aldrich (Saint Louis, Missouri, USA), and 3-Indoxyl sulfate-d4 potassium salt (IS-d4) purchased from Toronto Research Chemicals (North York, Ontario, Canada) was used as an internal standard.

### 2.2 Instrumentation

LC-MS/MS was performed using the AB SCIEX QTRAP® 4500 LC-MS/MS System, which is a unit in which an electrospray ionization system (ESI) and QTRAP and Triple Quad mass spectrometer are combined. The analyses were performed using MultiQunat™ software (AB SCIEX). As a comparator assay method for IS concentration, analysis was performed using an automatic biochemical analyzer H-7180 (HITACHI High-Tech Corporation) using an indoxyl sulfate assay reagent (Nipro Corporation, Osaka Prefecture, Japan).

### 2.3 Calibrators and quality control materials

The IS standard solution was preserved at −80 °C after preparation. Indoxyl sulfate potassium salt was adjusted to 300 mM with pure water. The solution of internal standard IS-d4 was adjusted to 1 mg/mL (3.917 mM) with acetonitrile solution, dispensed in small amounts, and preserved at −80 °C.

### 2.4 Sample preparation

The samples were sera from kidney disease patients at Chiba University Hospital who had provided informed consent. Eash serum was preserved at −80 °C until use. Serum with zero concentration IS was prepared by inactivating pooled serum with sulfatase. Test of the effects of interferents was performed using Interference Check A Plus (Sysmex, Kobe, Japan).

For the analysis, 50 μL of sample was mixed with 500 μL of 99.9 % acetonitrile solution containing IS-d4 at 3.917 mol/L, stirring the mixture for 30 s on a vortex mixer, and centrifuging it at 13400×*g* for 15 min. After centrifugation, 100 μL of the supernatant was mixed with 900 μL of pure water, and the mixture was assayed with the AB SCIEX QTRAP 4500 LC-MS/MS System. This study was carried out with approval by the ethical committees of Chiba University Hospital (677, 685, 1817) and International University of Health and Welfare (18-Io-186).

### 2.5 Chromatography and mass spectrometer (MS) conditions

IS was analyzed by the previously reported method [12]. The sample was injected onto a reversed-phase octadecylsilyl column, InertSustain C18 column (2.1 × 100 mm, particle size 3 μm, GL Sciences) at 40 °C. Purified water containing 0.1 % formic acid (pH 2.61) was used as mobile phase A, and acetonitrile containing 0.1 % formic acid (pH 3.17) was used as mobile phase B. The flow rate of the mobile phases was 0.45 mL/min, and the injection volume was 5 μL. The linear gradient was set as follows: 0–1 min, 80 % A; 1.0–1.7 min, 80 %–20 % A; 1.7–1.8 min, 20 %–5 % A; 1.8–3.4 min, 5 % A; 3.5 min, 80 % A. The analyte assayed was quantified using the AB SCIEX QTRAP 4500 LC-MS/MS System, which operates by negative electrospray ionization and in multiple reaction monitoring mode. The ion spray voltage was set at −4.5 kV, Ion Source Gas 1 at 50, and Ion Source Gas 2 at 80, and the source temperature was 300 °C. The precursor ion, product ion, cone voltage, and collision energy of the target analyte and internal standard were determined by directly injecting IS. The collision energy (CE) was −40 eV for IS and −30 eV for IS-d4. Transition of multiple reaction monitoring (MRM) was set as *m/z* 211.9 → 79.9, 211.9 → 131.9 for IS and *m/z* 215.9 → 79.9 for IS-d4 and optimized using MultiQunat™ software.

### 2.6 Method validation

The method was validated according to the guideline for validation of assay system of the Clinical & Laboratory Standards Institute (CLSI) or International Organization for Standardization [[14], [15], [16], [17], [18], [19], [20], [21]]. Tests other than the correlation test were performed by 2 examiners, and the inter-rater reliability was calculated. Calibration curves were constructed independently by each examiner for each measurement. A common internal standard was used. Quality control and equipment management were performed at each measurement. The intraclass correlation coefficient (ICC) was used as a measure of inter-rater reliability.

From the following equation:

#### 2.6.1 Linearity evaluation

Linearity was tested by referring to the procedure described in CLSI document EP06AE. Linearity was evaluated using the geometric mean regression (or standard major axis regression) method.

Specifically, dilution linearity using the standard solution and dilution linearity using the patient sample were examined. For the test using the standard solution, the IS standard indoxyl sulfate potassium salt (Sigma-Aldrich) solution at 300 μmol/L was used. A dilution series prepared by mixing the IS standard solution with pure water was assayed (Table 1). For the test of dilution linearity using the patient sample, a dilution series prepared by mixing the patient sample (IS level, 1.86 μmol/L) with the IS standard solution was assayed.

**Table 1:** 9-step dilution method.

Indoxyl sulfate concentration (%)	0	12.5	25	37.5	50	62.5	75	87.5	100
Indoxyl sulfate Standard solution concentration (μmol/L)	0	37.5	75	112.5	150	187.5	225	262.5	300
Indoxyl sulfate Standard solution + Patient serum concentration (μmol/L)	0	55	110	165	220	275	330	385	440

#### 2.6.2 Reproducibility testing

The repeatability (n = 20) and intermediate precision (n = 15) were examined using pooled serum adjusted to 3 concentration levels (repeatability: 9.8, 50.5, 125.3 μmol/L intermediate precision: 9.8, 50.5, 156.3 μmol/L). For intermediate precision testing, one of the samples preserved at −80 °C was thawed each day and examined. One sample of two series each was analyzed over 15 days.

#### 2.6.3 Correlation evaluation

Correlativity was evaluated using 42 samples from kidney disease patients at Chiba University Hospital. Indoxyl sulfate assay reagent (Nipro, Nipro Corporation, Osaka Prefecture, Japan) was used as a comparator for LC-MS/MS, and the 39-μmol/L standard solution accompanying the indoxyl sulfate assay reagent Nipro was used as the standard solution. Eight microliters of sample was preincubated with 160 μL of reagent A at 37 °C for 5 min, and the absorbance was determined at 450 nm (dominant wavelength) and 700 nm (sub-wavelength) using the automatic biochemical analyzer H-7180 (HITACHI High-Tech Corporation). Reaction was initiated by the addition of 40 μL of reagent B, and the absorbance was determined at 450 nm (dominant wavelength) and 700 nm (sub-wavelength) after 5 min. Pure water instead of the sample was used as a blank sample. A 50 μmol/L indoxyl sulfate potassium salt (Sigma) solution was used as a standard solution for LC-MS/MS. In addition, the calibration curve of LC-MS/MS was corrected using a standard solution for enzymatic assay. Regression analysis was performed by standard major axis regression.

#### 2.6.4 Selectivity tests

Selectivity tests were performed using 5 % human serum albumin (HAS) solution and serum (Table 2). To evaluate the matrix effect, the matrix factor (MF) was used [22,23].

**Table 2:** Samples used in the additive recovery study.

Sample No,	
1	normal saline solution
2	saline solution 9 parts, IS 2 mmol/L 1 parts
3	HAS buffer 9 parts, saline solution 1 parts
4	HAS buffer 9 parts, IS 2 mmol/L 1 parts
5	control serum 9 parts, saline solution 1 parts
6	control serum 9 parts, IS 2 mmol/L 1 parts

The matrix factor is calculated using the following formula:

MF(%)=PAs/PAr×100

IS normalized MF(%) = MFs/Mfis × 100.

MF(%) = Matrix Factor, IS = Internal Standard.

PAs = Peak area of the analyte or internal standard in the evaluation sample.

PAr = Peak area of the target sample.

MFs = MF of the analyte.

MFis = MF of the internal standard.

#### 2.6.5 Test of the effects of interferents

The effects of interferents were evaluated using Interference Check A Plus (Sysmex, Kobe, Japan). Each component of Interference Check A Plus (Sysmex, Kobe, Japan), including bilirubin F (F-Bil), bilirubin C (C-Bil), hemoglobin (Hb), and chyle, was prepared according to the manufactures’ instructions. To pooled patient serum, Interference Check A Plus (F-Bil, C-Bil, Hb, chyle) and the interferent blank corresponding to each component were added and mixed. Then, the mixture was assayed, and the change rates compared with the blank were calculated [24].

#### 2.6.6 Evaluation of the limit of blank (LOB), limit of detection (LOD), and lower limit of quantification (LLOQ)

Evaluation was made using non-IS water, and 5 samples each from healthy individuals. Non-IS serum was prepared by mixing 99 vol of pooled serum and 1 volume of sulfatase (activity: 1200 U/mL, Nipro Corporation, Osaka Prefecture, Japan) and incubating the mixture in a thermostat bath at 37 °C for 1 h. The samples were dispensed and preserved at −80 °C. In non-IS serum, the IS peak observed in pooled serum was confirmed to have disappeared. Each sample was dispensed to 2 vials, and each vial was assayed 3 times.

Limit of blank (LOB) was evaluated using pure water, physiologic saline, and non-IS serum as blank samples. Each of the 3 blank samples was assayed 4 times a day on 5 days, and data for a total of 60 assays (3 × 4 × 5) were obtained. Evaluation was made by calculating the grand mean (M_B_) and standard deviation (SDB) of the data.

Limit of detection (LOD) and lower limit of quantification (LLOQ) were evaluated using 5 samples from healthy individuals. Each of the 5 samples were assayed 4 times a day on 5 days, and the data for a total of 100 assays (5 × 4 × 5) were obtained. Combined standard deviation was calculated from the standard deviation of each sample, and the LOD was calculated by the following formula using the average standard deviation (SD_S_) [25].

$$\text{LOD}=\text{LOB}{+}_{\text{ZB}}\text{SDs}$$

Here, the coefficient ZB is the value at which the lower probability of standard normal distribution is B, and ZB = 1.65 when B = 0.05.

### 2.7 Statistical analysis

The statistical analyses were performed using Microsoft Excel and GraphPad Prism 9 (GraphPad Software, La Jolla, CA) software. LOB, LOD, and LLOQ were calculated using Validation-Support Ver.61 (Japan Society of Clinical Chemistry), which is a program for the validation of quantification methods.

## 3 Results

### 3.1 LC-MS/MS optimization

#### 3.1.1 Recording equilibration of MS

**Fig. 1:**
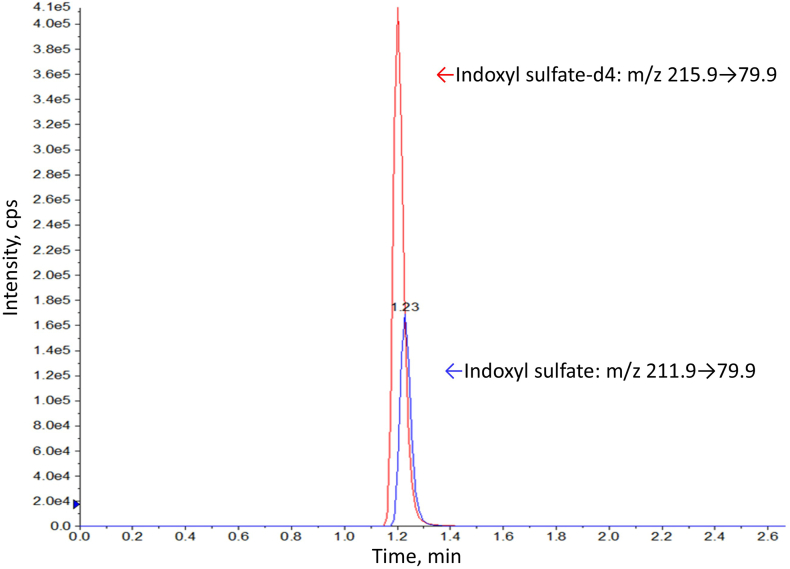
**Ion chromatogram.** Typical ion chromatogram obtained by analysis of indoxyl sulfate (IS), *m/z* 211.9 → 79.9, and 3-indoxyl sulfate-d4 potassium salt (IS-d4), *m/z* 215.9 → 79.9, from a control sample containing IS at 2 mg/L in serum matrix. The IS retention time was approximately 1.23 min.

Optimal internal parameters of MS were determined by directly injecting each target analyte at 100 ng/mL into the MS/MS detector. To optimize the mobile phases of chromatography, various concentrations of formic acid were tested. It was decided to use purified water containing 0.1 % formic acid as mobile phase A and acetonitrile containing 0.1 % formic acid as mobile phase B. The retention time of IS was about 1.2 min under each condition. The execution time was 5 min for each injection in baseline-resolved chromatographic separation. A typical ion chromatogram of the target analyte containing 50 μmol/L IS is shown (Fig. 1).

### 3.2 Method validation

Since the standard solution and all the samples were analyzed after being mixed with IS-d4 at the same concentration, the IS concentrations in the samples were calculated by preparing a calibration curve based on the target analyte (IS)/internal standard (IS-d4) peak area ratio in the standard solution.

#### 3.2.1 Linearity evaluation

**Fig. 2:**
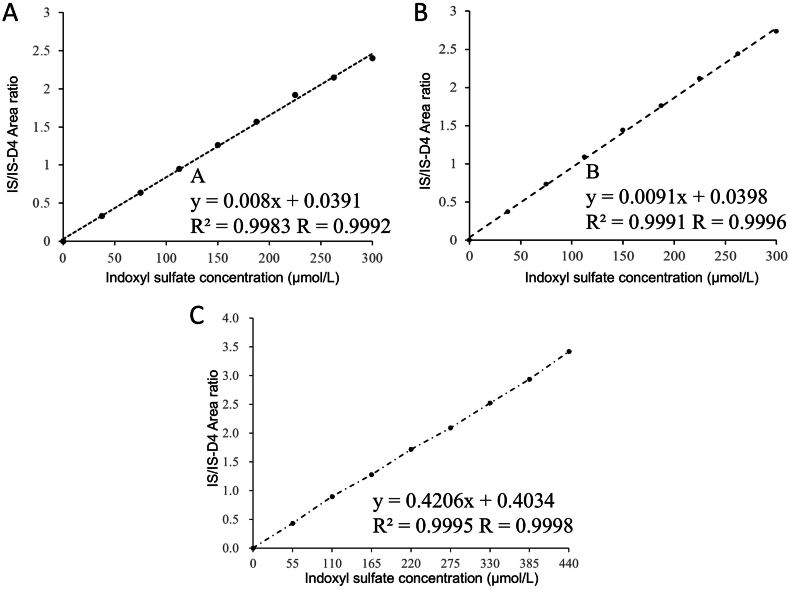
**Dilution linearity evaluation** Dilution linearity evaluated by examiner A using the standard solution is shown (A). Dilution linearity evaluated by examiner B using the standard solution is shown (B). Dilution linearity using patient serum is shown (C). Patient serum in which the IS concentration was determined by the enzymatic method in advance (1.85 μmol/L) was mixed with IS standard solution, and a concentration gradient was generated. The vertical axis represents the IS/IS-d4 peak area ratio. The horizontal axis represents the IS concentration.

Dilution linearity was tested separately by examiner A and examiner B. In consideration of the range of IS concentration in CKD patients and the concentration for clinical decisions, evaluation was made with 440 μmol/L as the maximum concentration. As a result, linearity passing through the origin was observed by both examiners A and B with the standard solution at 300 μmol/L (Fig. 2A and B) and the patient serum sample at 440 μmol/L (Fig. 2C).

#### 3.2.2 Test of precision of reproducibility

Regarding the repeatability, CV was 2.6–4.7 % for the mean of 8.3–124.6 μmol/L (Table 3). The same results were also obtained by examiner B. Moreover, the inter-rater reliability between examiners A and B according to intraclass correlation coefficient (ICC) was 0.999.

**Table 3:** Repeatability.

Examiner A	pooled serum concentrations (μmol/L)			Examiner B	pooled serum concentrations (μmol/L)		
	9.8	50.5	125.3		9.8	50.5	125.3
Ave (μmol/L)	8.3	50.9	123.6	Ave (μmol/L)	9.7	51.9	124.6
SD (μmol/L)	0.5	2.6	3.4	SD (μmol/L)	0.4	1.4	3.3
CV (%)	4.3	4.7	2.7	CV (%)	3.4	2.7	2.6

Regarding the intermediate precision, the IS concentration was determined by applying the value of IS/IS-d4 ratio obtained by LC-MS/MS to the equation used for the evaluation of dilution linearity. In the first series, the mean was 13.1, 59.4, and 170.4 μmol/L, SD was 1.1, 4.7, and 14.4 μmol/L, and CV was 8.2, 7.9, and 8.5 %. In the second series, the mean was 12.9, 59.8, and 171.2 μmol/L, SD was 1.0, 5.5, and 14.4 μmol/L, and CV was 8.0, 9.2, and 8.4 % (Table 4). The Control chart for assay stability is shown in Supporting information Figure S2. Quality Control samples were run during the analysis as part of a robustness evaluation.

**Table 4:** Intermediate precision.

Examination 1	pooled serum concentrations (μmol/L)			Examination 2	pooled serum concentrations (μmol/L)		
	9.8	50.5	156.3		9.8	50.5	156.3
Ave (μmol/L)	13.1	59.4	170.4	Ave (μmol/L)	12.9	59.8	171.2
SD (μmol/L)	1.1	4.7	14.4	SD (μmol/L)	1.0	5.5	14.4
CV (%)	8.2	7.9	8.5	CV (%)	8.0	9.2	8.4

#### 3.2.3 Correlation evaluation

**Fig. 3:**
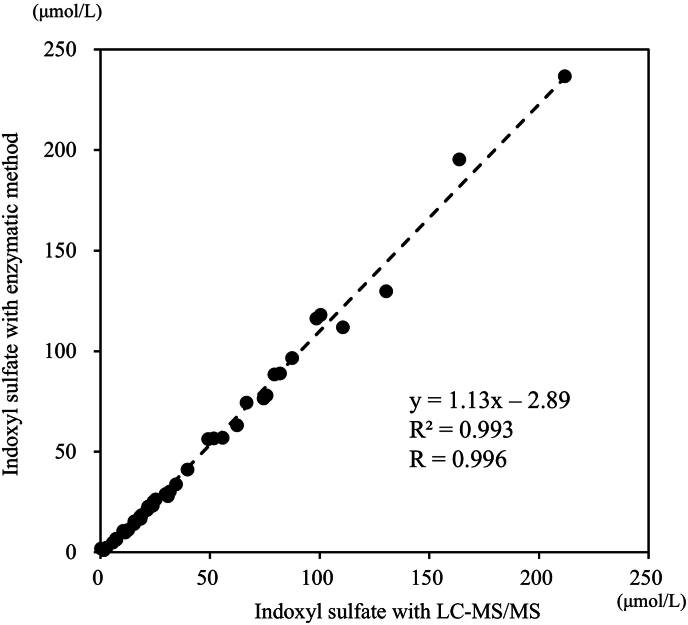
**Correlation Evaluation** The values by the enzyme method performed with an automatic biochemical analyzer are shown along the vertical axis. Those obtained by LC-MS/MS are shown along the horizontal axis.

The correlation between this method (y) and the comparator method (x) was y = 1.13x - 2.89, and the correlation coefficient was R = 0.996 (Fig. 3).

#### 3.2.4 Selectivity tests

On the selectivity test, the recovery rate was 101.1 % by examiner A and 101.0 % by examiner B for HAS buffer solution and 104.3 % by examiner A and 104.3 % by examiner B for the control serum (Table 5). On the Matrix Factor, the recovery rate was 101.7 % by examiner A and 100.6 % by examiner B for HAS buffer solution and 105.5 % by examiner A and 105.7 % by examiner B for the control serum (Table 5). Both CVs were less than 1.5 %. Saline solution was Not Detected.

**Table 5:**
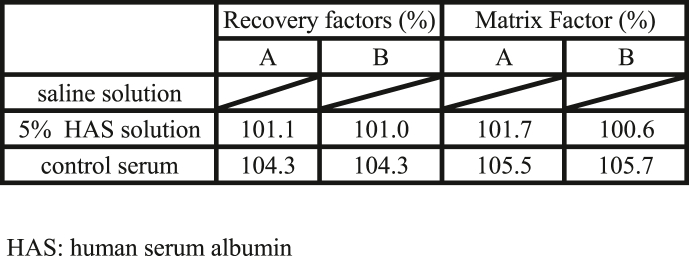
Selectivity Test.

#### 3.2.5 Test of the effects of interferents

By examiner A, the effects on the IS concentration were 5.4 % by F-Bil, 3.0 % by C-Bil, 0.0 % by hemolytic Hb, and 2.9 % by chyle. By examiner B, the effects on the IS concentration were 4.7 % by F-Bil, 1.0 % by C-Bil, 0.1 % by hemolytic Hb, and 2.6 % by chyle (Table 6).

**Table 6:** Influence of interfering substances.

Examiner A	IS concentration	Examiner B	IS concentration
	rate of change (%)		rate of change (%)
F-Bil (344 μmol/L)	5.4	F-Bil (344 μmol/L)	4.7
C-Bil (345 μmol/L)	3.0	C-Bil (345 μmol/L)	1.0
hemoglobin (4.5 g/L)	0.0	hemoglobin (4.5 g/L)	0.1
chyle (1770 FTU)	2.9	chyle (1770 FTU)	2.6

FTU: Forumajin Turbidity Units.

#### 3.2.6 Evaluation of the limit of blank (LOB), limit of detection (LOD), and lower limit of quantification (LLOQ)

LOB, LOD, and LLOQ calculated from the concentrations measured by examiners A and B are reported. LOB was 0.000 by the parametric method and 0.004 by the non-parametric method by both examiners A and B. Concerning limit of decision (CCα), the concentration calculated by examiner A was 1.14 μmol/L and that calculated by examiner B was 0.66 μmol/L [26]. Concerning LOD, combined standard deviation of the IS/IS-d4 ratio calculated by examiner A was 0.0043, 0.0071 by the parametric method, and 0.0111 by the non-parametric method. Concerning the concentration, the combined standard deviation was 0.76, 1.244 μmol/L by the parametric method and 1.248 μmol/L by the non-parametric method. By examiner B, the combined standard deviation of the IS/IS-d4 ratio was 0.0029 and 0.0047 by both the parametric and non-parametric methods. Concerning the concentration, combined standard deviation was 0.45 μmol/L and 0.745 μmol/L by both the parametric and non-parametric methods.

**Fig. 4:**
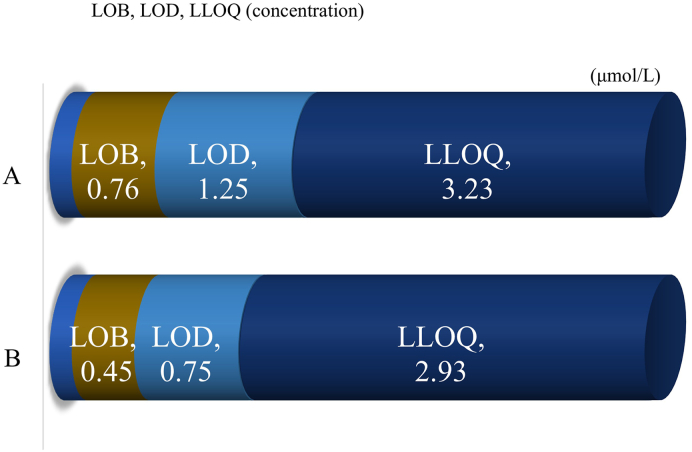
**LOB, LOD, LLOQ Evaluation** IS concentrations reported by examiners A and B are shown at the top and bottom, respectively.

By examiner A, LLOQ was 0.083 and 0.01 when CV of IS/IS-d4 was 10 % and 20 %, respectively, and the concentration was 73.49 μmol/L and 3.23 μmol/L, respectively. By examiner B, LLOQ was 0.019 and 0.00 when CV of IS/IS-d4 was 10 % and 20 %, respectively, and the concentration was 2.93 μmol/L and 0.00 μmol/L, respectively (Fig. 4). To confirm that the LLOQ met the required threshold according to FDA and EMA guidelines, a representative chromatogram is shown in Fig. S1.

## 4 Discussion

In this study, a method for the assay of indoxyl sulfate (IS) using LC-MS/MS was established. Then, its performance was evaluated according to the FDA guidelines or CLSI C62-A [14]. The use of an internal standard is important in liquid chromatography mass spectrometry to ensure its precision. In this study, commercial IS-d4 was selected as an internal standard. The mass number of IS-d4 is 215.9 *m/z* while that of IS is 211.9 *m/z*, and IS-d4 did not interfere with mass spectrometry. Regarding the mobile phases, the optimization was validated by using purified water containing 0.1 % formic acid as mobile phase A and acetonitrile containing 0.1 % formic acid as mobile phase B.

In the linearity evaluation, the upper limit of quantification of IS concentration was set at 440 μmol/L in view of the clinical introduction. As a result, dilution linearity passing through the origin was observed. In the precision testing, the repeatability and intermediate precision were satisfactory with CV of less than 4.7 % and 9.2 %, respectively. In the correlation evaluation, the assay results were compared between the enzymatic method using an automatic biochemical analyzer and LC-MS/MS, and a high correlation with R = 0.996 was indicated. LC-MS/MS showed no deviation from the conventional method and was validated as a reference method. The Bland Altman analysis is shown in Supporting information Figure S3. We used the Bland Altman analysis to determine systematic bias between LC-MS/MS and traditional enzymatic methods. Since proportional error was recognized, a percent difference plot was performed. Accurate detection was possible in low indoxyl sulfate. We believe that the lower correlation at higher indoxyl sulfate concentrations is clinically acceptable. On the selectivity testing, the recovery rate was 101.0–104.3 %, and no interference was noted. On the interference testing, slight errors were observed in F-Bil, but it was about 5.0 % at the maximum. Regarding the limit of blank (LOB), limit of detection (LOD), and lower limit of quantification (LLOQ), the results concerning the no-detection zone, indeterminant zone, detection zone, and quantification zone were in agreement between examiners A and B. In addition, concerning LLOQ, the minimum concentration was calculated in the permissible range with CV of 20 % by examiner A and with CV of 10 % by examiner B. Since the acceptable error range calculated from the mean values in the quantification zone using the acceptable error limits recommended by the Japan Society of Clinical Chemistry [25] was (3.23 + 2.93)/2 ± 5 % = 3.08 ± 0.15 μmol/L, the variation observed in this study due to interrater disagreement is considered to be in the acceptable range [27].

In recent analytical chemistry, diversification of assay targets, lowering of assay concentration, and greater accuracy are increasingly demanded. With sophistication analytical instruments, operation manuals have been developed and improved, allowing anyone to calculate accurate values regardless of the degree of proficiency of the examiner. In clinical laboratory testing, various tests have been standardized, and adjustments to obtain accurate results are now possible [28,29]. There are necessary conditions for the standardization of clinical laboratory tests, and one of them is the presence of a reference material and a reference testing procedure. This study was conducted to establish a reference internal standard and reference method for the accurate assay of indoxyl sulfate as a biomarker of CKD. We hope that the results obtained in this study lead to the standardization of clinical testing of indoxyl sulfate.

In summary, an assay method for IS using LC-MS/MS could be established. In addition, it was proved that the effects of interferents on the IS assay method using LC-MS/MS are extremely small. The method made consistent calculation of LOB, LOD, and LLOQ. The IS assay by LC-MS/MS using IS-d4 as an internal standard has the potential to contribute to the standardization of clinical testing as a reference method.

## CRediT authorship contribution statement

**Sohei Kobayashi:** Writing – original draft, Conceptualization. **Kazuyuki Matsushita:** Writing – review & editing. **Kenji Kawasaki:** Data curation. **Masanori Seimiya:** Project administration, Funding acquisition.

## Funding

This work was supported by 10.13039/100018504Nipro Co., Ltd.

## Declaration of competing interest

The authors declare the following financial interests/personal relationships which may be considered as potential competing interests: Sohei Kobayashi reports equipment, drugs, or supplies was provided by Nipro Co., Ltd. If there are other authors, they declare that they have no known competing financial interests or personal relationships that could have appeared to influence the work reported in this paper.

## Figures and Tables

**Figure:**
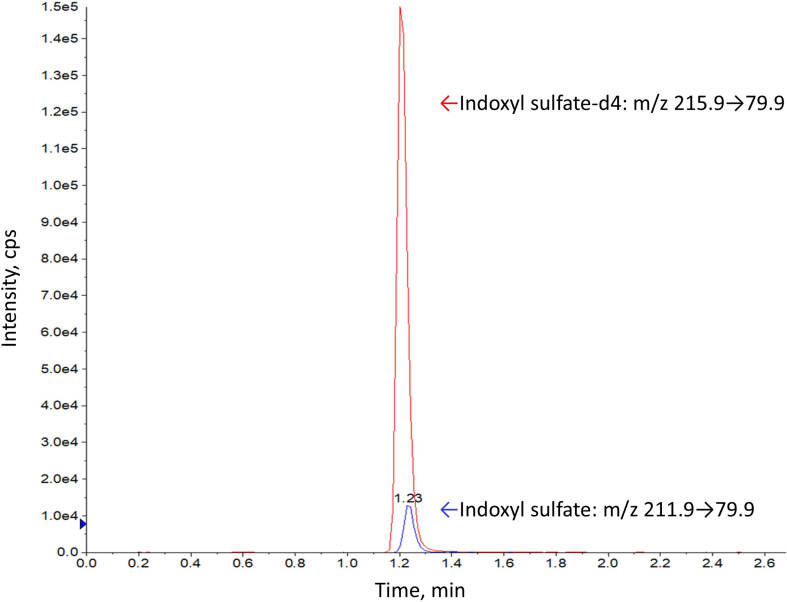
Supporting information Figure S1. Ion chromatogram of LLOQIndoxyl sulfate (IS), *m/z* 211.9 → 79.9, and 3-indoxyl sulfate-d4 potassium salt (IS-d4), *m/z* 215.9 → 79.9, were analyzed. The IS retention time was approximately 1.23 min.

**Figure:**
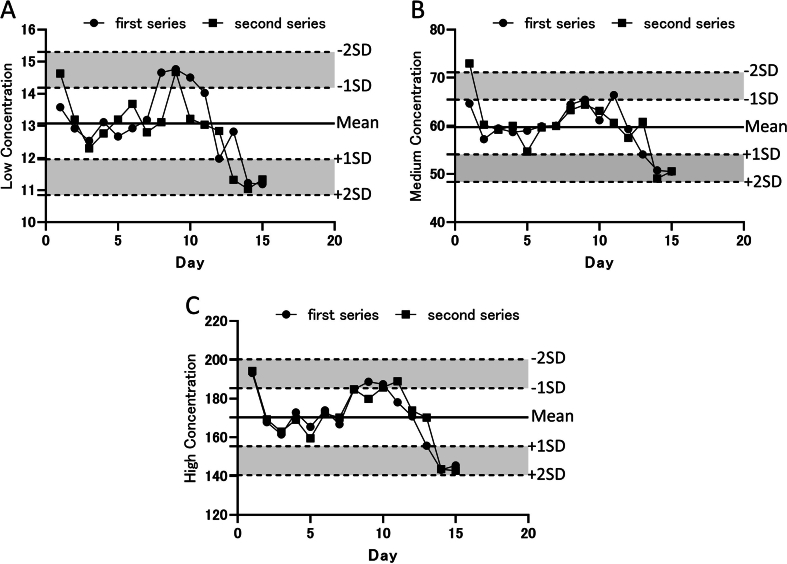
Supporting information Figure S2. The Control chart for assay stability We used the Levey–Jennings charts to determine the control chart for assay stability. In this Levey–Jennings chart, statistical analyzes were performed using GraphPad Prism 9 (GraphPad Software, La Jolla, CA) software. One sample of two series each was analyzed over 15 days. (A) The Levey–Jennings chart for low concentrations is shown. (B) The Levey–Jennings chart for medium concentrations is shown. (C) The Levey–Jennings chart for high concentrations is shown.

**Figure:**
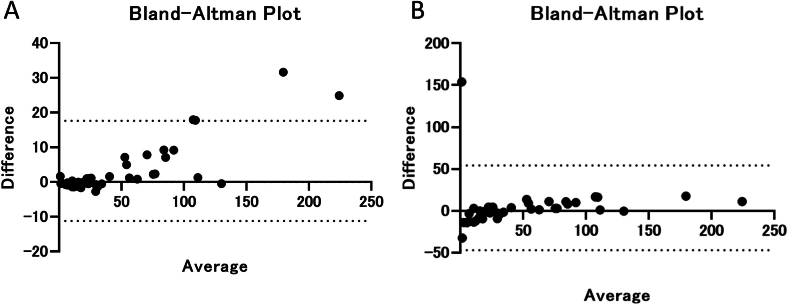
Supporting information Figure S3. Bland Altman analysis We used the Bland Altman analysis to determine systematic bias between LC-MS/MS and traditional enzymatic methods. In this Bland Altman analysis, statistical analyzes were performed using GraphPad Prism 9 (GraphPad Software, La Jolla, CA) software. (A) Bland Altman plot. (B) Percent Difference plot.

## Data Availability

Data will be made available on request.
