# Supplementary material for: Clinical Quantitative Method Development of serum indoxyl sulfate (IS) assay using LC-MS/MS
Source: Biochem Biophys Rep. 2025 Oct 24;44:102316. doi: 10.1016/j.bbrep.2025.102316 (PMC12593637; doi:10.1016/j.bbrep.2025.102316)
Supplement: Multimedia component 1 [file mmc1.docx]

**AUTHOR CHECKLIST *Authors of all papers should submit this checklist plus the checklist from the relevant reporting guideline together with their manuscript.*** *Part 1* identifies basic requirements for the manuscript submission *(mandatory for all submissions)*

*Part 2* identifies recognized guidelines for scientific reporting, which you should use to prepare your manuscript *(required for systematic reviews and original research)*

| ***PART 1 Basic requirements*** | | ***Author response or further detail – please complete the boxes below*** | |
| --- | --- | --- | --- |
| *Word count* | | 3492 | |
| *Was ethical approval given and by whom? (give any reference number)* | | This study was carried out with approval by the ethical committees of Chiba University Hospital (677, 685, 1817) and International University of Health and Welfare (18-Io-186). | |
| *Please state any conflicts of interest* | | The authors declare the following financial interests/personal relationships which may be considered as potential competing interests. This work was performed in collaboration with Nipro Co., Ltd. | |
| *Please state sources of funding and the role of funders in the conduct of the research* | | This research did not receive any specific grant from funding agencies in the public, commercial, or not-for-profit sectors. | |
| *Please state any study registry number (e.g. ISRCTN)* | | This study is not a clinical trial and is not registered. | |
| ***For the items below, please tick in the right hand column to confirm you have included/addressed the items:*** | | | **Tick** |
| *Title* | Confirm that the title is in the format ‘Topic / question: design/type of paper’ and identifies the population / care setting studied.  *(e.g. The effectiveness of telephone follow-up in managing symptoms after breast cancer radiotherapy: a randomised controlled trial).* The structure is optional for discussion papers, editorials and letters) | | Tick |
| *Abstract* | A structured abstract appropriate to the design of the study is included (see *guidelines for authors)*. | | Tick |
|  | No references are cited in the abstract. | | Tick |
| *Key words* | Between four and ten key words have been provided in alphabetical order, which accurately identify the paper's subject, purpose, method and focus. Use the Medical Subject Headings (MeSH®) thesaurus or Cumulative Index to Nursing and Allied Health (CINAHL) headings where possible (see <http://www.nlm.nih.gov/mesh/meshhome.html>). | | Tick |
| *Highlights* | Bullet points have been included that identify existing research knowledge relating to the specific research question / topic (what is already known about the topic?) and a summary of the new knowledge added by this study (what this paper adds) *(see Guide for Authors*, does not apply to editorials or letters) | | Tick |
| *Abbreviations* | No abbreviations are used in the title / abstract. Use of abbreviations /acronyms in the paper is minimised and restricted to those that are likely to be universally recognized (e.g. USA) | | Tick |
| *References* | All citations in the paper have a complete and accurate reference in the reference list (see *Guide for Authors)* | | Tick |
| Other Published accounts | *All* published and in press accounts of the study from which data in this paper originate are referred to in the paper and the relationship between this and other publications from the same study is made clear (see *Guide for Authors*) (Please upload copies of all previous, current and under review publications from this study and / or give full details below) | | Tick |
|  | Please provide references of ANY other papers using data from the study that this paper is based on) below. | | Tick |
|  | The study is referred to by a distinctive name which will be used in any future publications to identify that it is the same study (e.g. RN4Cast) | | Tick |
| *Authorship* | All authors and contributors sufficiently acknowledged as per Guide for Authors. | | Tick |

| ***PART 2***  ***Standards of reporting*** | The editors require that manuscripts adhere to recognized reporting guidelines relevant to the research design used. These identify matters that should be addressed in your paper. Authors of research papers and systematic reviews are required to submit a checklist relevant to the research design they have used. The checklist will be drawn on within the peer review process. Please indicate which guideline (below) that you have referred to and ensure that the relevant checklist is uploaded.  These are not quality assessment frameworks and your study need not meet all the criteria implied in the reporting guideline to be worthy of publication in the EJON. The checklists do, however, identify essential matters that should be considered and reported upon. For example, a controlled trial may or may not be blinded but it is important that the paper identifies whether or not participants, clinicians, outcome assessors and analysts were aware of treatment assignments. | **Checklist submitted^[[1]](#footnote-1)^**** |
| --- | --- | --- |
| Observational cohort, case control and cross sectional studies | STROBE **St**rengthening the **R**eporting of **Ob**servational Studies in **E**pidemiology  <http://www.equator-network.org/reporting-guidelines/strobe/>   |  |
| Quasi experimental / non-randomized evaluations | TREND - Transparent Reporting of Evaluations with Non-randomized Designs <http://www.cdc.gov/trendstatement/>   |  |
| Randomised (and quasi-randomised) controlled trial | CONSORT – Consolidated Standards of Reporting Trials  <http://www.equator-network.org/reporting-guidelines/consort/>   |  |
| Study of Diagnostic accuracy / assessment scale | STARD Standards for the Reporting of Diagnostic Accuracy studies  <http://www.equator-network.org/reporting-guidelines/stard/>   |  |
| Systematic Review of Controlled Trials | PRISMA - Preferred Reporting Items for Systematic Reviews and Meta-Analyses  <http://www.equator-network.org/index.aspx?o=1032>   |  |
| Systematic Review of Observational Studies | MOOSE Meta-analysis of Observational Studies in Epidemiology  <https://www.elsevier.com/__data/promis_misc/ISSM_MOOSE_Checklist.pdf>   |  |
| Qualitative studies | Standards for Reporting Qualitative Research (SRQR)*  <http://www.equator-network.org/reporting-guidelines/srqr/>   | Tick |
| Clinical Practice guidelines | The AGREE Reporting Checklist: a tool to improve reporting of clinical practice guidelines  <http://www.equator-network.org/reporting-guidelines/the-agree-reporting-checklist-a-tool-to-improve-reporting-of-clinical-practice-guidelines/>   |  |
| Other (please give source) | Please check <http://www.equator-network.org/> for other designs or other sources |  |
| Not applicable (please elaborate) |  |  |

1. [↑](#footnote-ref-1)
